# Improved adjuvant endocrine therapy for premenopausal women with endocrine responsive disease

**DOI:** 10.3332/ecancer.2015.544

**Published:** 2015-06-09

**Authors:** Aron Goldhirsch, Marco Colleoni, Meredith Regan

**Affiliations:** 1 Program of Breast Health (Senology), European Institute of Oncology, Via Ripamonti 435, Milan 20141, Italy; 2 Division of Medical Senology, European Institute of Oncology, Via Ripamonti 435, Milan 20141, Italy; 3 Department of Biostatistics and Computational Biology, Dana-Farber Cancer Institute, 450 Brookline Avenue, Boston, MA 02215, USA; 4 International Breast Cancer Study Group (IBCSG), Effingerstrasse 40, Bern 3008, Switzerland

**Keywords:** adjuvant endocrine therapy, breast cancer, SOFT & TEXT, ovarian function suppression, exemestane

## Abstract

Results from two randomised global trials (SOFT & TEXT) designed to newly define the most effective components of adjuvant endocrine therapy for premenopausal women with endocrine responsive disease, showed that for some, those with high risk of relapse, the use of the aromatase inhibitor exemestane together with ovarian function suppression with GnRH analogue (triptorelin) yielded the most favourable treatment outcome compared with tamoxifen. For women with low risk of relapse, treatment with tamoxifen was similar to ovarian function suppression together with either exemestane or tamoxifen. For women with intermediate risk of relapse, ovarian function suppression added to tamoxifen was not inferior to exemestane, while it resulted in superior outcomes compared to tamoxifen alone. Now, these trials provide critical information for the adjuvant treatment of premenopausal women with endocrine responsive breast cancer and are important for the development of future trials for further improvement of adjuvant endocrine therapies for the younger population.

## Content

Adjuvant endocrine therapy for premenopausal patients with endocrine responsive breast cancer includes tamoxifen given either after (neo) adjuvant chemotherapy or immediately after surgery, without previous chemotherapy, either alone (mostly a US and Australian standard) or with ovarian function suppression for 2–5 years using GnRH analogue (mostly European standard). Trials in advanced disease indicated that the combination of GnRH analogue and tamoxifen is superior in terms of clinical efficacy compared with each of the two treatments alone [1, 2]. SOFT and TEXT are complementary trials designed for global conduct in countries in which tamoxifen alone (after chemotherapy or immediately after surgery) was considered a standard of care (SOFT with 3,066 patients) or in which combined tamoxifen and GnRH analogue was considered standard of care (TEXT with 2672 patients). Both trials had common treatment options (GnRH analogue with tamoxifen or with exemestane), while SOFT also had a tamoxifen-alone control. Premenopausal patients with endocrine-responsive disease could have either chemotherapy followed by the randomly assigned endocrine therapy or start immediately after randomisation with the randomly assigned endocrine therapy. Another important difference between SOFT and TEXT was the timing of start of the assigned endocrine therapy, that is, in TEXT, GnRH analogue started immediately after randomisation, which occurred early, before start of chemotherapy, if planned, and in SOFT, the time lag to start of assigned endocrine therapy was longer for patients who received post-operative chemotherapy. These patients had to wait for verified premenopausal status (biochemically, if no recent menses) after chemotherapy to be certain about the planned ovarian function suppression with GnRH analogue (assigned to two-thirds of the patients).

The results of the trials are extensively described in two recent papers published in the New England Journal of Medicine [3, 4]. The data were first analysed for presentation at a median follow-up time of about 68 months, a time frame that is appropriate to assess early treatment effects in an endocrine responsive population but is far from indicating long-term treatment efficacy. The most striking results derived from the combined analysis of SOFT and TEXT on treatment effect of patients who had ovarian function suppression with either tamoxifen or exemestane. Disease-free survival at 5 years was 91.1% in the exemestane ovarian function suppression group and 87.3% in the tamoxifen ovarian function suppression group (hazard ratio = 0.72; 95% confidence interval CI: 0.60–0.85; *P* < 0.001).

Results from SOFT for patients who remained premenopausal after completing chemotherapy indicated substantial improvements in 5-year disease-free survival for women assigned to exemestane-ovarian function suppression (83.8%) or to tamoxifen-ovarian function suppression (80.7%) compared with tamoxifen alone (77.1%) (exemestane-ovarian function suppression versus tamoxifen hazard ratio = 0.70; 95% CI: 0.53–0.92; tamoxifen versus tamoxifen-ovarian function suppression hazard ratio = 0.82; 95% CI: 0.64–1.07).

Adding ovarian suppression to tamoxifen increased the adverse events that patients experienced, most notably menopausal symptoms, but the adverse event profiles of exemestane-ovarian function suppression versus tamoxifen ovarian function suppression were similar to those observed for aromatase inhibitors (AI) versus tamoxifen in postmenopausal women. Patients self-reported differences with respect to specific symptoms from the three treatments, but the overall quality-of-life assessment did not favour any of the three treatments. The most interesting development after the publication of the two trials was related to the St. Gallen consensus panel deliberations [5]. The panel considered treatment recommendations for two clinical scenarios. The first involved a 42-year-old patient with node-negative, grade 2, T1, ER-positive tumour not receiving chemotherapy. A large majority of the panel members would assign tamoxifen alone as adjuvant systemic treatment. The second scenario involved a 34-year-old patient with lymph node-positive, grade 3, T1, ER-positive disease who remained premenopausal after adjuvant chemotherapy. The advised treatment includes ovarian function suppression combined with exemestane rather than tamoxifen for this patient.

## Conclusion

The panel considered that factors arguing for the inclusion of ovarian function suppression are, in case of young age (35 or less), persisting premenopausal oestrogen level after adjuvant chemotherapy, or involvement of four or more axillary nodes. Fewer panel members considered grade-3 disease or an adverse result from a multiparameter molecular marker test as indications for ovarian function suppression (with either tamoxifen or exemestane).

Future developments in the field will include the following:

1. The definition of a risk scale to help in the choice of proper endocrine therapy components.
2. Treatment efficacy of the various endocrine therapy components during a longer follow–up.
3. Attempt to improve the yield of ovarian function suppression by the use of GnRH antagonists and the addition of drugs which enhance the efficacy of endocrine agents such as those recently described in advanced disease for postmenopausal women (e.g., CDK 4/6 inhibitors).
